# “I just don’t know enough”: Australian perspectives on community involvement in health and medical research

**DOI:** 10.1186/s40900-024-00633-8

**Published:** 2024-11-28

**Authors:** Fiona Russo, Isabella Sherburn, Keri Finlay, Jack Nunn, Monica Ferrie, Anne McKenzie, Sean Murray, John Cannings, Greg Pratt, Tiffany Boughtwood

**Affiliations:** 1 Centre for Health Research, University of Southern Queensland, Toowoomba, QLD Australia; 2 Australian Genomics, Murdoch Children’s Research Institute, Melbourne, VIC Australia; 3 Science for All, Melbourne, VIC Australia; 4 Genetic Support Network of Victoria, Melbourne, VIC Australia; 5 Telethon Kids Institute, Perth, WA Australia; 6 Community Advisory Group, Australian Genomics, Melbourne, VIC Australia; 7 Mito Foundation, Sydney, NSW Australia; 8 Office of Indigenous Engagement, Central Queensland University, Rockhampton North, QLD Australia

**Keywords:** Community, Genomic research, Involvement, Australia, Perspectives, Engagement

## Abstract

**Background:**

There is increasing global support from governments and other funding bodies for community involvement in research, alongside a scientific and moral imperative for responsible and ethical research practice. Ninety per cent of Australian patient-led organisations in rare diseases have clearly articulated research priorities, indicating a desire among people affected by disease to be involved in research that impacts their communities. Philanthropic research, which is likely to have predominantly community-minded priorities, is worth over AU$1 billion annually and increased more than 100% between 2007 and 2017.

**Aims:**

This research aimed to understand public perspectives on community involvement in health-related research activities, and to inform the development of guidelines for genomic researchers to improve this involvement.

**Methods:**

A 37-question survey was completed by 1,156 members of the Australian public via Dynata. The survey was co-designed by the Involve Australia Working Group of community members within Australian Genomics. Results from 1156 responses were analysed.

**Results:**

Key themes emerging from the survey data that impact potential involvement were low community confidence to contribute, a limited understanding of community involvement, roles and recognition, trust and governance of data, perceived trustworthiness of research funders, and factors related to time and personal resources. A variety of motivations for involvement were also stated.

**Conclusion:**

Members of the Australian public are interested in research involvement; however the differences between involvement and participation are poorly understood and a variety of barriers still exist. Researchers must actively reach out into communities and offer opportunities to engage with research and identify community priorities.

**Supplementary Information:**

The online version contains supplementary material available at 10.1186/s40900-024-00633-8.

## Abstract (plain language summary)

It is well understood that involving the community in research about them is an important part of good science, and it helps to make sure that health research has a positive impact on communities (people who use the health services).

This study aims to understand how the Australian public feels about becoming involved in research about their community’s health.

A team of community members and researchers from a group called Involve Australia designed a survey to ask members of the Australian public to share their views. The survey was sent out by a company called Dynata and 1,156 people responded.

• More than half of the people said they were living with a health condition
• One third of the people said that they lived with someone who had a health condition
• One third of the people said they had university degrees
• Most of the people had Australian ancestry
• Under one third of the households earned more than $100,000 per year

Only 26 people had been involved in research before, and from these people:

• 18 had a university degree
• Just over half of the people were working in paid jobs
• More than half also worked as volunteers
• Half of the households earned more than $100,000 per year

All members of the Involve Australia team helped to analyse the survey results for all 1,156 people, which showed us that many people did not understand the difference between involvement and participation. We know this because lots of people said they would not get involved with research because they were worried about their private information being shared or scared of the medicines or procedures (for example, needles) they might have to take. These reflect activities of participation, not involvement.

A quarter of the people were worried they did not have enough knowledge or education to become involved in research. They were not confident that they could add value to research.

Some people (15%) said they weren’t interested in research, but the ones who said they were interested told us they wanted to help with designing the research, helping to write the research papers, and updating their communities on the research. They also said they wanted their work to be recognised and they would like to be paid for their time, and that they would be more likely to get involved if it was easy. For example, they spoke about not travelling a long way, using Zoom and phone, not paying for parking, and making sure the places they had to go were accessible for everyone.

People said they were more likely to get involved if they trusted the researcher or the company doing the research. They were more likely to get involved if the organisation was not a private company trying to make money.

Most of the people said they would get involved in research that helped someone they loved, or people in their community. Many told us that they wanted younger people with health issues to have a better life than they had, and that they would get involved with research to make that happen.

This research shows that community members and researchers should speak with each other more often. People said they wanted to know what research was happening and they also wanted to tell researchers what they thought was most important.

The Involve Australia group suggests that research institutions and universities need to have good relationships with the people in their communities, and that researchers need to have time and resources to build these relationships.

**Supplementary Information:**

The online version contains supplementary material available at 10.1186/s40900-024-00633-8.

## Introduction

### Community involvement in health research

Robust community involvement ensures that research is impactful, and supports its effective translation into health service delivery. It is the role of government and industry to ensure social and ethical responsibility is prioritised [1]. There is increasing global support for community involvement in research from governments and other funding bodies, where engagement reporting is part of the grant assessment criteria. One such example is the Canadian Institutes of Health Research which has adopted the term ‘citizen engagement’ to describe community-led or co-designed research [2]. In the Australian context, a variety of toolkits, guidelines, frameworks, and statements demonstrate the growing commitment of governments and funding bodies toward including the ‘voice’ of the public, communities, and consumers in research [3]. Australia’s largest medical research funding body, the National Health and Medical Research Council (NHMRC), highlights community engagement as a requirement on some funding applications, and there are actions underway to extend this across all areas of research.

Biomedical researchers from the US, UK, Africa, and Australia indicate strong support for community engagement in research, citing ethical imperatives such as empowerment and instrumental and transformative goal setting [4]. The UK’s James Lind Alliance [5] brings together caregivers, patients, and clinicians in priority setting partnerships that align funding rounds to community needs. The National Institute of Health in the US requires all funded clinical trials to involve community members in the research design [6]. These all suggest that community-initiated and synergistic models of research that reflect the tenets of ‘citizen science’ and forefront the principles of collaboration and co-design are well placed to prioritise the community voice.

### Involve Australia

This study, led by Involve Australia, seeks to understand the gap between the broadly positive intent of funders, institutes, researchers and clinicians to engage with community, and actual community involvement in genomics research. We surveyed 1,156 Australians about their views on health research and community involvement and asked them about their experiences and/or intention to become involved themselves. Involve Australia is a community-led project coordinated by Australian Genomics and is informed by a diverse working group that includes patient advocates (JC, FR), patient support and advocacy group leaders (MF, SM), involvement experts (AM, JN) and researchers (TB, KF, GP, IS, FR), collaborating to give the public a stronger voice in genomic research and its translation into clinical practice. Involve Australia aims to inspire and enable people to be involved meaningfully in all parts of genomic research by bringing stakeholders together to optimise research outcomes. A primary outcome of this research is the development of community involvement guidelines for genomic researchers [7], within the Australian context.

### Definitions

In this research, Involve Australia has adopted a definition of ‘community’ aligned to the NHMRC, which describes a community as:*“a group of people sharing a common interest (e.g. cultural, social, political, health, economic interests) but not necessarily a particular geographic association”* and acknowledges that diverse communities are likely to have differing perspectives and approaches to their involvement in research [8].

 Involve Australia has further adopted the following definitions from the Australian Clinical Trials Alliance [9]:

- Involvement: when consumers and community representatives actively work with researchers and research organisations to help shape decisions about health research priorities, policy, and practice.
- Engagement: when information and knowledge about research is shared to better inform consumers and the community on why, how, where and by whom research is conducted.
- Participation: where an individual voluntarily takes part in a research project after giving informed consent.

### Community involvement in genomics research

Since the sequencing of the entire human genome in 2003, genomic research has significantly advanced our understanding of human physiology and facilitated the development of diagnostics, medicines, and therapies for a variety of health issues at the DNA level. Human genomics, defined as the study of the complete set of genetic instructions and how these interact with each other and the environment [10, 11] also makes possible practices that are ethically complex, such as human cloning and genetic discrimination, and require community involvement in decision making. It is forecast that more than 60 million individuals internationally will have their DNA sequenced by 2025 [12]. In this rapidly evolving landscape, it is critical that community members are meaningfully and responsibly involved at all levels of research, from governance and priority setting to project design and knowledge dissemination [13].

Improving how communities are involved in genomic research can be considered both a scientific and moral imperative, necessary for responsible and ethical research practice. In addition, for genomic research to be successful, it requires both public support for funding, and a willingness from people to participate and give consent to share data [14, 15]. Community involvement is particularly important with respect to populations at greater risk of exploitation, such as those with histories of experiencing medical and research abuse, including Indigenous peoples [16].

Several countries have demonstrated the often life-changing benefits of genomics in rare diseases and cancers [17]. In 2016, 90% of Australian patient-led organisations in rare diseases had clearly articulated research priorities [18]. Despite reporting significant challenges in meeting these goals, more than 95% of the 112 surveyed organisations had undertaken at least one research-related activity, such as providing funding to researchers, in the preceding five years. Patterson, O’Boyle, VanNoy & Dies [19] later surveyed 159 patient advocacy groups in the United States and found that 79% were involved in research and 81% listed research engagement as a top priority. This clearly demonstrates a desire among people affected by rare diseases to be involved in research that impacts their communities. Significantly, private ‘not-for-profit’ research (such as charity-funded research), which is likely to have predominantly community-minded priorities, is worth over AUD 1 billion annually and has increased more than 100% between 2007 and 2017 [20]. Despite this, patient advocacy groups report misalignment of researcher and community priorities, a lack of genuine involvement in decision-making [18], varying levels of researcher and collaborator investment, and limited supports and training for community representatives [9] as significant barriers to involvement.

With the growing recognition of the importance of community involvement in genomics research evident in community members, researchers, and funding bodies, we should expect to be seeing far more involvement (and reporting of involvement) in current literature, yet this is not the case. Hoekstra et al. [21] looked at 86 literature reviews that analysed approximately 870 individual primary studies mentioning community partnership in all health research and found that only 18 of the 86 reviews contained detailed information on the engagement of stakeholders at different phases of the research process. While engagement of stakeholders was referenced, 15 of these 18 reviews noted a lack of consistent reporting with high levels of variability in the detail provided about the methods, breadth, depth, and evaluation of community involvement. Similarly, Esmail, Moore and Rein [22] reported a ‘striking’ lack of consistent assessment or evaluation of consumer engagement in their own literature review on stakeholder engagement in research. More recently the Global Alliance for Genomics and Health has suggested ‘ways of conducting evaluations of engagement’, including recommending the use of models such as Standardised Data on Initiatives (STARDIT) and Guidance for Reporting Involvement of Patients and the Public (GRIPP2) checklist [12]. As genomic sequencing becomes more widely available and accessible to people with and without suspected/identified genetic disorders, it is important to understand public perspectives on community involvement in research activities.

### Research Aim

This study aims to identify public sentiments related to health research involvement, which will inform the development of guidelines for genomic researchers to improve community involvement practices.

## Methods

This study was informed by the participatory methodologies first described by Freire and Ramos [23] and later refined for community-based health research [24] and genomics research specifically [25, 26].

Detailed information on how different stakeholders (including community members and researchers) were involved in the Involve Australia project can be found in the associated STARDIT report [27].

### Co-Design in the study

The Involve Australia Working Group developed an initial online survey which was reviewed by a plain language advisor to ensure it was accessible to a broad audience. The tasks of the Involve Australia Working Group were to progress Involve Australia activities by providing input and advice on methods used to conduct the survey and involve the community effectively. Working group members attended monthly formal meetings via videoconferencing. These meetings informed members of project updates and provided time for discussion and feedback on key documents, which were also made available outside meetings for review. For this study, members contributed to survey development and dissemination, data analysis, and drafting of this research output.

### Survey design

The survey was designed to collect the perspectives of the Australian public on being involved in health research as community representatives. It included an exploration of the perceived factors influencing involvement and reflections on previous experiences with health research. The survey was piloted with 55 respondents prior to a full launch to ensure a clear understanding of the questions. One question which had been identified as causing some confusion in the pilot was reworded, but no major revisions were made.

The final survey (Supplementary Material 1) consisted of 30 multiple choice questions and seven (including four “other” text boxes) open text response questions. Demographic data collection accounted for 21 of the 37 questions.

Questions referred to health research more generally, rather than genomics research, as we anticipated this would increase misunderstandings as Little, Koehly & Gunter [28] reported that the public continue to struggle with the concept of ‘genomics’. However, we believe that responses to questions about health research involvement more generally can still inform practices for community involvement in genomics research.

### Recruitment

Survey respondents were recruited via an internationally recognised contracted market research provider. Dynata [29] recognises local privacy and data protection laws and maintains Australian certification with the Research Society Fair Data Accreditation (certificate number ISOEX-110011-2). People were eligible to complete the survey if they were over 16 years of age and residing in Australia. Parental consent was obtained for participants between 16 and 17 years of age. The survey was also distributed by several patient support and advocacy groups to recruit adult Australians who had been involved as a community member in health research as we wanted some data on those who had been involved in research as community members and we anticipated this may be a limitation of Dynata’s broader public recruitment.

### Analysis

Quantitative data were imported into the statistical analysis software STATA 17 [30]. This software was used to carry out descriptive statistical analysis of categorical variables, such as whether respondents were living with a health condition, and the demographic information collected. The relationship between multiple variables was analysed using cross tabulation, and Pearson’s chi-square tests were used to establish whether differences were significant.

Qualitative (open response) data was imported into NVivo software [31] and analysis was conducted to identify key themes and notable outliers. Up to ten clear themes emerged from each question with superordinate themes present in the collected responses across the entire survey. The stages of qualitative data analysis included data mapping and familiarisation; transcription; coding; searching for themes; reviewing themes with study team members; labelling and summarising themes; and reporting the findings. Qualitative themes were identified by the first author (FR) and checked for validity by two authors (KF, IS).

## Results

In total, 1206 members of the public responded to the survey. During initial review 50 responses were removed as they provided nonsense or inconsistent responses. The remaining 1156 responses were analysed. The following data was drawn from those 1156 responses.

Of the participants, 51% were female with a mean/median age range of 35–44 (Table 1). All demographic information was self-reported. The presence of a health condition was reported by 51% of the participants (*n* = 591) and 36% indicated that they lived with someone with a health condition (*n* = 411). Details of the health condition were not collected.

Of the total respondents, 27 individuals (2%) indicated that they have taken part in health research as a community member. One response was removed as they subsequently commented in an open text response that they had not been involved in health research as a community member, therefore 26 responses were analysed.

Females represented 54% (*n* = 14) of the community members who indicated prior involvement in research. Community members were predominantly highly educated with 69% (*n* = 18) holding an undergraduate or postgraduate qualification. This is greater than what was indicated in the broader cohort where 37% of individuals held an undergraduate or postgraduate qualification. Most community members (73%) indicated they were of Australian ethnicity, which was similar to the general cohort (78%). Marginally more community members were in paid employment (54%, *n* = 14), working > 30 h a week (50%, *n* = 7). Fifty-three per cent (*n* = 14) of community members volunteered in some capacity. Notably, this was greater representation than was seen in the general cohort where 24% of people regularly volunteered. Household income for community members was predominantly above $100,000 with 50% earning over this threshold. In the general cohort, 31% of respondents had a household income over $100,000. A vast majority of community members are living with a health condition (88%, *n* = 23), which is much higher than the general cohort where 51% reported living with a health condition (*n* = 591) (Table 1).

**Table 1:** Relevant Respondent demographics

Variables	Attribute	Full cohort (*n* = 1156)		Community members involved in research (*n* = 26)	
		Freq	Percent	Freq	Percent
Gender	Female	587	50.8	14	53.9
	Male	565	48.9	12	46.1
	Other	4	0.4	0	0
Age	16–17	10	0.9	0	0
	18–24	127	11	3	11.5
	25–34	194	16.8	4	15.4
	35–44	229	19.8	4	15.4
	45–54	215	18.6	2	7.7
	55–64	182	15.7	5	19.2
	65–74	134	11.6	3	11.5
	> 75	65	5.6	5	19.2
Education	< Yr 12	173	15	0	0
	High School	183	15.8	2	7.7
	TAFE/ Certificate	362	31.3	5	19.3
	Undergraduate	287	24.8	7	26.9
	Postgraduate	144	12.5	11	42.3
	Chose not to answer	7	0.6	1	3.9
Ethnicity*~	Australian	904	78.2	19	73.1
	British	81	7	4	16.4
	Chinese	52	4.5	0	0
	Italian	28	2.4	0	0
	Indian	14	1.2	0	0
Income (AUD)	<$20K	64	5.5	1	3.9
	$20–34 K	148	12.8	4	15.4
	$35–49 K	147	12.7	1	3.9
	$50–74 K	204	17.7	1	3.9
	$75–99 K	160	13.8	3	11.5
	$100–149 K	227	19.6	10	38.5
	>$150K	127	11	3	11.5
	Nil income	10	0.9	0	0
	Chose not to answer	69	6	3	11.5
Aboriginal and/ or Torres Strait Islander Ethnicity	Aboriginal	38	3.3	1	3.8
	Torres Strait Islander	2	0.2	0	0
	Aboriginal and Torres Strait Islander	4	0.3	1	3.8
Living with a health condition ^+^	Yes	591	51.1	23	88.5
	No	565	48.9	3	11.5
Living with someone with a health condition^+^	Yes	411	35.6	12	46.2
	No	745	64.5	14	53.8

*Top five self- reported ethnicities, ~More than one ethnicity could be selected, ^+^ Some overlap between people with a health condition and people living with someone with a health condition

**Fig. 1:**
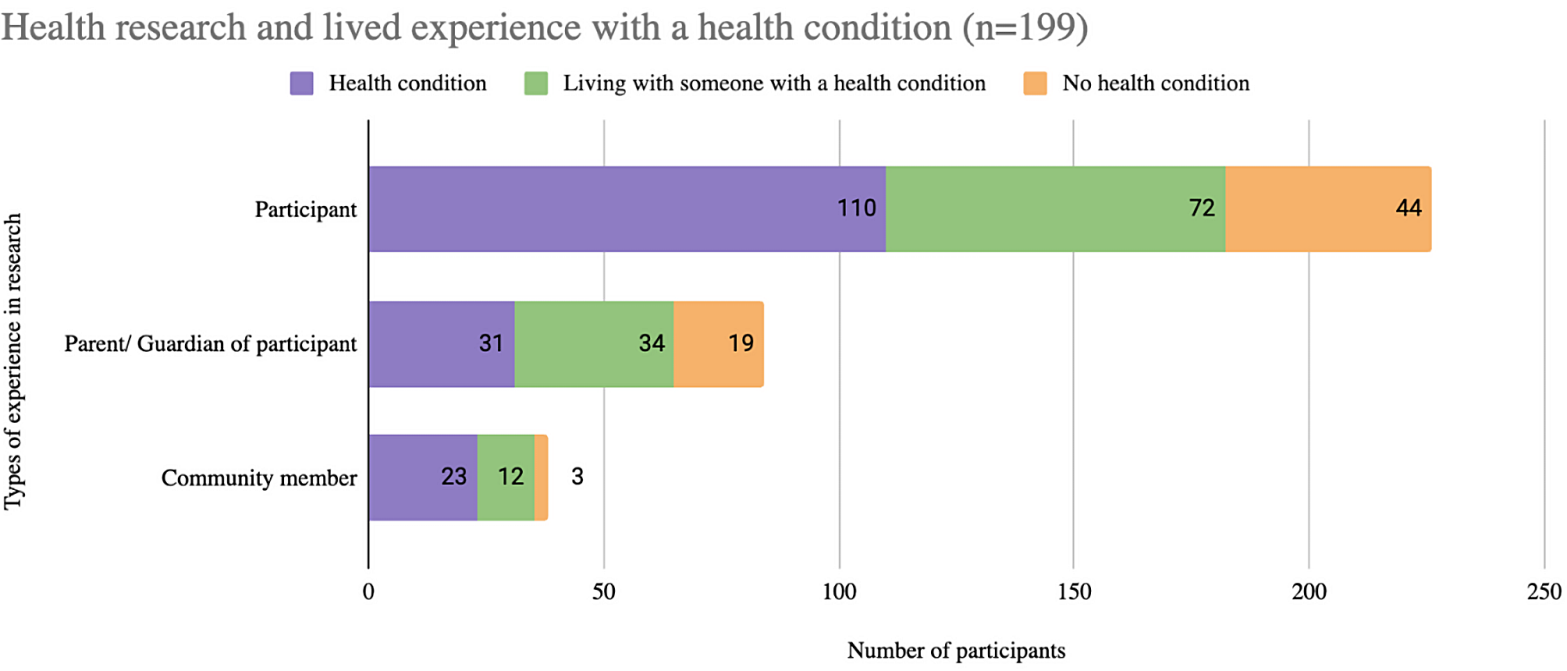
Research experience and health conditions. *Some overlap between people with a health condition and people living with someone with a health condition

Respondents were able to select multiple choices. Among the 1156 respondents, 17% (*n* = 199) reported previous experience with health research, with 77% (*n* = 154/199) people engaged as participants and 25% (*n* = 50/199) as parents or guardians of a participant. 71% (*n* = 110/154) of research participants also indicated that they have a health condition. A small percentage (3%, *n* = 35/1156) of individuals who did not report a health condition or living with someone with a health condition had taken part in health research as a participant (*n* = 27/35) or parent or guardian of a participant (*n* = 8/35). Of this group, two individuals had taken part as a participant and a parent or guardian (Fig. 1).

**Fig. 2:**
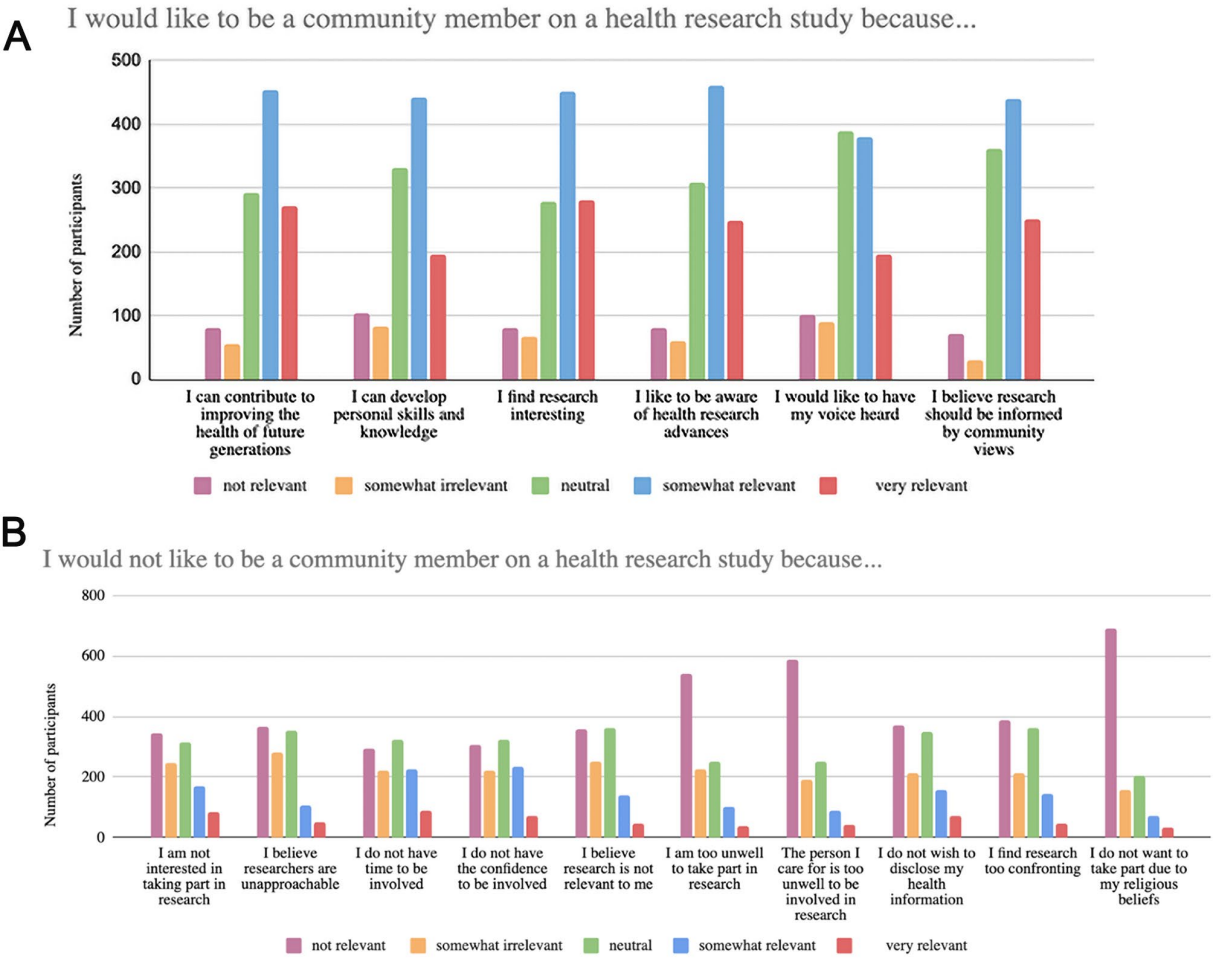
(**A** and **B**): Factors influencing community involvement in research

### Mixed methods analysis

Multiple choice responses relating to community perceptions of being involved in health research (Fig. 2) are supported by superordinate themes present in the responses to open text questions. Key themes from both and representative of all respondents are presented here.

### Involvement vs. participation

Answers relating to personal health risk and data privacy concerns were common. Even though questions used the term **community involvement**, respondents were clearly considering the collection and storage of personal medical data and samples and the trialling of therapies and medications in open text responses. These are all examples of participation activities. This issue was also noted in the Likert scale answers, where we asked respondents why they *would not* want to be involved in research and 20% (*n* = 227) indicated that they had concerns about sharing their private health information. This points to an enduring and fundamental misunderstanding of community involvement vs. participation in research studies.

### Respondent confidence

Another key theme related to individual respondent confidence. Many respondents indicated concern about the perceived value of their potential contributions to research. Comments related to respondents’ age, education level, and whether their contributions would be ‘scientific enough’ to be useful to researchers, with comments such as *“I may not provide relevant expertise”,* *“[I don’t know] whether my input would be of value”,* and *“what would I be able to contribute personally?”* among the responses. Across the cohort, 26% (*n* = 305) respondents cited a ‘lack of confidence’ in their ability to contribute positively to research.

### Respondent roles and recognition

 Fifteen per cent of survey respondents said they had not been involved in research because they were not interested, perhaps indicating a lack of personal investment in health research. This is not altogether surprising as the presence of a health condition is likely to indicate a personal investment in health research, further supported by the previously described proportion of respondents with personal or family health concerns. Notable alongside the discussion of respondent confidence was the perceptions of the role/s community members can and should have in health research. One respondent said, *“what does it really mean, like what’s my role?”* Respondents indicated a variety of preferences when asked how they ‘would like to be involved’ in research, including research translation (42%, *n* = 481), reviewing communication documents (43%, *n* = 494), knowledge dissemination (40%, *n* = 467), outcomes measures (39%, *n* = 447), and participant recruitment (33%, *n* = 383). The most positively associated roles are in research design (43%, *n* = 496), contributing to research outputs (47%, *n* = 538), and research updates (59%, *n* = 683). This supports responses (29%, *n* = 340) which further indicated that they would be more likely to become involved if their input was acknowledged: [I want to be] *“recognised as a researcher or included in academic outputs”;* [I want] *“recognition, pay, and to be interested in the research”;* [I want] *“my contributions to be recognised”*.

### Trust and governance

Some interesting findings include the prevalence of open text responses relating to trust - trust in the organisation and/or individuals conducting the research, the funding source, and the drivers for research. Respondents expressed a desire to understand who was doing the research and why, and particularly where the money was coming from. Responses suggested that public institutions and community-led organisations would be considered trusted partners as their own motivations were perceived as ‘right’ or ‘good’, but private institutions with obvious commercial interests were likely to be viewed with more suspicion.

### Motivation

Many respondents discussed the direct benefit to themselves, their family members, or their communities as reasons to take part in research at any level. Others spoke about improving the health outcomes of younger people with their diagnoses or positive outcomes for others with the same/similar conditions as primary motivators for involvement. Many responses indicated both personal and community level benefits as motivators. Notably, comments indicating ‘for the greater good’ altruistic intent outweighed those motivated by personal benefit. This motivation was also evident in the Likert scale responses, where 63% (*n* = 727) of respondents said they would choose to become involved in research to ‘contribute to the health of future generations’(Fig. 2).

### Time, remuneration and accessibility

Overall, results suggest that while people have generally positive attitudes towards involvement in research, concerns around time commitment, recognition of value in the form of remuneration, and research accessibility persist as barriers. Comments such as [I would need to consider] *“the costs associated for myself”,* *“cost or remuneration for taking part”,* and *“I would expect to be paid for my time”* demonstrate a desire among respondents to be recompensed for their time and contributions. Additionally, many identified an inclusion issue, reporting that they would be unable to get involved as they could not afford the time, or conflicting work or caring responsibilities. At the least, there is a clear expectation that community representatives should not bear any financial costs for research involvement: 62% (*n* = 717) of respondents indicated they would be more likely to become involved if they were ‘paid for any transportation costs to and from meetings’; and 63% (*n* = 723) said that being ‘paid for my [their] time/role’ would increase the likelihood of involvement. 45% (*n* = 261) also indicated they would want ‘researchers to organise and fund any services I [they] need to take part’.

Many respondents expressed concern about the time and effort required for the research involvement. Open text comments such as *“I don’t have enough time free”,* *“I have other commitments”,* and [I would need to consider] *“time and distance constraints”,* and *“time and cost to me”* suggest that while respondents are broadly supportive of involvement, research is not their core business and must be accommodated in their day-to-day lives. When asked why they would not get involved, 27% (*n* = 316) of respondents said they ‘do not have time to become involved’.

Accessibility was another key theme, but a clear definition of ‘accessible’ research was not evident. For example, 31% (*n* = 359) of respondents said they would prefer ‘meetings are face-to-face’ while 47% (*n* = 543) said they would prefer ‘meetings are conducted online’. No consensus on preferred meeting format was evident. Carefully designed involvement activities that are mindful of the burden to community members might also reduce barriers for the respondents who indicated that the ‘person I [they] care for’ (11%, *n* = 128) or they themselves (12%, *n* = 138) are ‘too unwell’ to become involved in research.

### Communication

This key theme highlights the importance of clear and open communication with community members about the time and activities required for involvement, as well as the project itself - the risks, benefits, outcomes, and governance structures. However, the theme of communication was not purely researcher-community member but also included intra-community communication, with some respondents expressing concern about how their involvement might be viewed by their partners, families, and broader communities, for example stating that they would need to consider *“how my community would perceive me”* before involving themselves in research.

Families were mentioned in open text responses in three main contexts: (a) impact on time and family duties; (b) direct benefit of the research for family members; and (c) the support of families and partners as a consideration in committing time and energy to research involvement.

### “Community”

The understanding and interpretation of the term ‘community’ was another interesting finding. Comments indicated a range of attitudes toward ‘community’, from the uncertain: *“I don’t even know what the [sic] community member is”;* *“I’ve no idea what being a community member involves”,* to the dismissive: *“not being part of a community, I wouldn’t feel right being involved”;* *“I am not part of any community at all”;* *“I don’t have time to participate in the community”,* and even expressing negative feelings about it: *“I do not like the community I live in at all”;* *“I am not involved with my community and don’t want to be”;* *“I don’t want other members of my community knowing anything about me”*.

## Discussion

This paper sought to understand the perceptions of the Australian public toward becoming involved in health research as a community member. Many open text responses suggested that there was some confusion between being a ‘participant’ and being a ‘community member’ who is involved, despite definitions and examples being provided in the survey. Qualitative answers suggest respondents were certainly thinking about being a participant rather than an involved community member, which implies that the public think about research involvement primarily in terms of research participation. Although there is a wealth of research looking at ways to improve general health literacy among consumers [32–35], health *research* literacy is rarely included. As a relatively new field of research, genomics complicates this further. Little, Koehly & Gunter [28] report that the public continue to struggle with the concept of ‘genomics’, despite improvements in the US publics’ familiarity and understanding of genetics from 2013 to 2021. Without initial improvements in health research literacy, involving the community in genomics research will continue to be restricted to those who have lived experience despite genomic testing becoming more widely accessible.

Enablers of community involvement identified included remuneration and reimbursement of expenses for community members. We posit that related barriers such as the amount of time and effort required to complete involvement activities, accessibility (geographic, structural, and physical) of research, and concerns about the value or usefulness of people’s contributions may explain the finding of homogeneity among the people who reported they had been previously involved in research. These were predominantly wealthy, well-educated people of Australian ancestry, and as such are more likely to be able to manage the costs of involvement (time and money), to have confidence about the value of their contributions, and to feel culturally and linguistically comfortable/safe. Lack of diversity in community members could further contribute to health disparities between communities, particularly in genomics where there is currently an over-representation of genetic data from those of European ancestry leading to genomic medicine having reduced applicability in non-Europeans [36, 37]. This finding of homogeneity supports prior studies that highlighted a need to improve the representation of marginalised individuals and communities [38, 39] and further suggests that valuing community member contributions financially and offering increased flexibility in the activities themselves could improve the diversity among involved community members by making research more accessible for all. Mentions of self-confidence among the reasons community members would not be likely to become involved in research are aligned with findings from previous studies [40]. When considering the increased likelihood of health issues among a community group such as those invested in genomics research, issues of time, effort, and confidence may be compounded by existing medical and therapeutic commitments and personal resource limitations (physical and psychological). The lack of consensus about meeting format coupled with expressed concerns about the costs of transportation and services would suggest that flexible involvement is the ideally accessible format to facilitate the inclusion of marginalised voices.

Issues relating to trust were evident in the open text responses, where transparent sharing of research funding sources, aims, motivations, benefits, and outcomes was highlighted as a facilitator of trust between researchers and community members. This supports the findings of Passmore et al. [41], which demonstrated a strong appetite among the public for building community knowledge rather than seeing research getting stuck in journal articles where it would be largely inaccessible to consumers. Trust is further cemented when researchers and community members discuss sensitive handling and reporting of patient data [42].

The responsibility for improving the understanding of health research must be shared among researchers, clinicians, administrators, educators, and the community. It is not a problem to be solved by one party who must ‘impose’ health research literacy on the populace. The authors of this paper join with research colleagues who advocate for closing the gap between professional and scientific health literacy and ‘lay’ literacy by using plain language summaries and infographics [43–47], publishing in accessible places [38, 48–51], and describing and acknowledging the involvement of community members [21, 52] in our research output. These activities would naturally strengthen general researcher-community connections, the importance of which was identified in this study.

Relationship-building within the community must exist beyond the scope of individual studies to build trust and increase communication. Researchers must engage with communities using open and transparent communication and meeting people in their own space, e.g. community events and centres. To facilitate this, research institutes must recognise and adequately resource the building of community relationships through mutually beneficial activities.

## Conclusion

Members of the Australian public are interested in being involved in research, however current community involvement is sporadic, unstructured and poorly reported. Researchers must reach out more actively into the community to offer opportunities to engage with research and identify community priorities [53] and better engage with the communities they serve when participatory research opportunities are identified – with research aims and benefits clearly communicated. If these relationships were stronger, there may be more interest in community involvement in research activities which would in turn improve the research itself and provide more community benefit, thus strengthening the relationship further. Research would better reflect the priorities of the communities and with stronger levels of investment, collaboration, and education, community members could themselves develop and lead research studies, recruiting research expertise where needed. The gap between scientific knowledge and general health literacy should not be a barrier to this engagement. For example, no expertise is required to comment on ethical issues such as data storage/access.

Australian people are interested in being involved but there are noted barriers to this, key amongst them communication, knowing how to be involved, self-confidence, time and personal resources. Current research does not report consistently about community and stakeholder engagement practices. This study highlights the need for more standardised, high quality community involvement in health research. Robust guidelines such as those developed by Involve Australia [7] can inform a consistent, evidence-based approach, promote and support community involvement, and provide a roadmap toward best practice among Australian health research communities. Resourcing community engagement activities at the institute level will empower researchers to foster robust community connections, develop impactful research priorities with communities, promote trust, and decrease tokenism in involvement activities and reporting. This will build confidence in funding bodies to recognise genuine involvement in health research funding applications. We join with our colleagues in asking funders to consider mandatory involvement and reporting of community involvement in the awarding of public research funds.

### Limitations

We acknowledge that data may be skewed by using a market research company that provides payment for completion. Overall, the survey respondents did not include a large sample of the public who had been involved in health research as community members. We additionally acknowledge the small sample of respondents from a non-English speaking background but suggest that this reflects overall homogeneity in community involvement.

### Future research

We suggest the inclusion of more children and young people’s voices. This survey was designed for a respondent cohort aged 16 and above, but the involvement of children and young people is critical to paediatric health and genomics research. Additionally, noting that the prevalence of particular language, socioeconomic status, and education demographics among respondents in this study mirrors that of community involvement more broadly, we suggest that further research on how to involve communities whose voices are not currently being heard would be valuable.

## Electronic supplementary material

Below is the link to the electronic supplementary material.

Supplementary Material 1

Supplementary Material 2

## Data Availability

Data is held by Australian Genomics and may be made avialable upon request. It is not publicly archived due to limitations of consent.
