# Supplementary material for: “I just don’t know enough”: Australian perspectives on community involvement in health and medical research
Source: Res Involv Engagem. 2024 Nov 28;10:126. doi: 10.1186/s40900-024-00633-8 (PMC11603817; doi:10.1186/s40900-024-00633-8)
Supplement: Supplementary file 1 — Supplementary Material 1 [file 40900_2024_633_MOESM1_ESM.docx]

**Health Research and You**

**Part 1: Information about you**

**In this section, we ask about what things in your life impact your interest and ability to take part in health research. These are things like your age, sex and gender, level of education and so on. By health research, we mean research that helps improve people’s health and wellbeing. Health research also aims to improve health services and their delivery.**

- 1. **I live in Australia** *required
  - Yes
  - No [Terminate]
- If this is selected thank the participant for their time, but they are unable to complete the survey
  1. **My age is…** (one choice only) *required
- <16 years of age
  - If this is selected thank the participant for their time, but they are unable to complete the survey
- 16 - 17 years of age
- 18 – 24 years of age
- 25 – 34 years of age
- 35 – 44 years of age
- 45 – 54 years of age
- 55 – 64 years of age
- 65 – 74 years of age
- 75+ years of age
  1. **I have a health condition**
- Yes
- No
  1. **I live with someone who has a health condition**
- Yes
- No
  1. **My biological sex is…** (one choice only)
- Male
- Female
- Intersex
- Prefer not to say
  1. **My identified gender is…** (one choice only)
- Male
- Female
- Other [open text box]
- Prefer not to say
  1. **My highest level of education is…** (one choice only)
- Year 10 or below
- Year 11
- High school diploma
- Trade certificate
- Associate Diploma
- Certificate II
- Advanced Diploma
- Undergraduate degree
- Post graduate degree
- Doctorate
- Prefer not to say
  1. **My postcode is** [open text box] (if possible, only allow entry of four numbers)
- Prefer not to say
  1. **My ethnicity is…** (multiple selections allowed)
- Australian

**1.9.1** Are you of Aboriginal or Torres Strait Islander origin?

No

Yes, Aboriginal

Yes, Torres Strait Islander

Yes, both Aboriginal and Torres Strait Islander

Prefer not to say

- Chinese
- British
- German
- Irish
- Italian
- Jewish
- Other [open text box]
- Prefer not to say
  1. **The languages I speak at home include** (multiple selections allowed)
- English
- Arabic
- Cantonese
- Greek
- Italian
- Mandarin
- Vietnamese
- Other [open text box]
  1. **Including myself, ____ people currently live in my home** (one choice only)
- 1
- 2
- 3
- 4
- More than 4
  1. **____ number of people in my household are under the age of 18** (one choice only) [question is hidden for respondents under 18 years of age]
- 0
- 1
- 2
- 3
- More than 3

- 1. **I have paid employment**
- Yes
  - 1. **I work ______ per week**
    - Less than 5 hours
    - 5-9 hours
    - 10-19 hours
    - 20-29 hours
    - 30-39 hours
    - 40 hours or more
    - Prefer not to say
    1. **My job title is** _____ [open text box]
  - Prefer not to say
- No
- Prefer not to say
  1. **I do volunteer work**
- Yes
  - 1. **I do volunteer work ______ per week**

Less than 5 hours

5-9 hours

10-19 hours

20-29 hours

30-39 hours

40 hours or more

- Prefer not to say
  - 1. **What area do you volunteer in?** _____ [open text box]
  - Prefer not to say
- No
- Prefer not to say
  1. **I do ____ of unpaid household work (e.g. caring for children, doing laundry, preparing meals) per week**

Less than 5 hours

5-9 hours

10-19 hours

20-29 hours

30-39 hours

40 hours or more

- Prefer not to say
  1. **The income group my household falls under is…** (one choice only)
- Less than $20,000
- $20,000 to $34,999
- $35,000 to $49,999
- $50,000 to $74,999
- $75,000 to $99,999
- $100,000 to $149,999
- $150,000 or more
- Nil income
- Prefer not to say

**Part 2: Taking part in Health Research**

**We would like to know if you have taken part in health research. Health research, helps improve people’s health and wellbeing. Health research also aims to improve health services and their delivery. We are interested in how you were involved and what encouraged you to participate. If you have not taken part in health research, we would like to know why. We would also like to know if you would like to take part in future health research.**

- 1. **I have been involved in health research** **(as a participant, parent/guardian of a participant, or as a community member on a health research study)** (one answer only)
- Yes
  - 1. **I was involved as a…** (multiple choice allowed)
    - Participant (e.g. answering surveys, providing blood samples)
    - Parent/guardian of a participant (e.g. answering surveys, providing blood samples)
    - Community member on a health research study (e.g. involved in research design, advising on research tasks) [leads to 2.1.2]
    1. **Health research activities I was involved in included** (multiple choice allowed) [this question only appears if ‘community member on a research study’ is selected above]
- Providing my opinion on the design of a research project (e.g. developing the project aims and outcomes)

Talking to researchers about how to get participants on board

Discussing with researchers how to measure success of a project

- Getting updates on how the research is going
- Discussing with researchers how to share the research with the community
- Reviewing project documents to share with the community
- Advising how research findings can be put into action
- Other [open text box]
  - - No
    1. **I have not been involved in health research (as a participant, parent/guardian of a participant, or as a community member on a health research study) because** (multiple choice allowed)
    - I am not interested in taking part in research
    - I did not know I could be involved
    - I have not been asked to be involved
    - I do not have time to be involved
    - I do not have the confidence to be involved
    - Research is not relevant to me
    - I/the person I care for is too unwell to be involved in research
    - I do not wish to share my health information
    - I did not want to take part due to my religious beliefs
    - Other [open text box]
  1. **Someone I know has been involved in health research as a: (multiple choice allowed)**
- Participant (e.g. answering surveys, providing blood samples)
- Parent/guardian of a participant (e.g. answering surveys, providing blood samples)
- Community member on a health research study (e.g. involved in research design, advising on research tasks)
- I’m unsure
- No one I know has been involved in health research [Exclusive]

In the following questions (from 2.3 – 2.9) we will be asking what may impact your decision to take part as a **community member on a health research study**. This means you may help health researchers with the design of a project or other research tasks.

- 1. **Please rate the relevance of the following statements:
     I would like to be a community member on a health research study because…**  **[**Likert scale, 1 (not relevant at all), 2 (somewhat irrelevant), 3 (neutral), 4 (somewhat relevant) to 5 (very relevant)]
- I can contribute to improving the health of future generations
- I can develop personal skills and knowledge
- I find research interesting
- I like to be aware of health research advances
- I would like to have my voice heard
- I believe research should be informed by community views
- Other [open text box]
  1. **Please rate the relevance of the following statements.
     I would not like to be a community member on a health research study because… [**Likert scale, 1 (not relevant at all), 2 (somewhat irrelevant), 3 (neutral), 4 (somewhat relevant) to 5 (very relevant)]
     - I am not interested in taking part in research
     - I believe researchers are unapproachable
     - I do not have time to be involved
     - I do not have the confidence to be involved
     - I believe research is not relevant to me
     - I am too unwell to take part in research
     - The person I care for is too unwell to be involved in research
     - I do not wish to disclose my health information
     - I find research too confronting
     - I do not want to take part due to my religious beliefs
     - Other [open text box]
  2. **Reason/s I may be uncertain about being involved as a community member on a research health study include:** [open text box] (can be left blank)
  3. **Please rate the following statements.
     I am more likely to take part as a community member on a health research study if…** [Likert scale, 1 (very unlikely), 2 (unlikely), 3 (neutral), 4 (likely) to 5 (very likely)]
- I am paid for my time/role in the research
- I am paid for any transportation costs to and from face-to-face meetings
- I am recognised as a researcher or included on academic outputs
- Meetings are face-to-face
- Meetings are conducted online
- Researchers organise and fund any services I may need to take part (e.g. language interpreter, note-taker)
  1. **Please rate the following statements.
     As a community member on a health research study, I would like to be involved in…** [Likert scale, 1 (very unlikely), 2 (unlikely), 3 (neutral), 4 (likely) to 5 (very likely)]
- Providing my opinion on the design of a research project (e.g. developing the project aims and outcomes)

Telling researchers how to get participants on board

Discussing with researchers how to measure success of a project

- Getting updates on how the research is going
- Discussing with researchers how to share the research with the community
- Reviewing project documents to share with the community
- Advising how research findings can be put into action
- Contributing to research outputs
  1. **When deciding whether I would like to take part as a community member on a health research study, I also consider:**
     [open text box] (can be left blank)
  2. **I would also like the researchers to know the following about taking part as a community member on a health research study:** [open text box] (can be left blank)

**Part 3: Being involved in future Involve Australia research**

- 1. **I would like to be contacted about future research involvement opportunities (e.g. reviewing project documents) with the Involve Australia project**
- Yes

**3.1.1 Activities I would like to take part in include:** (multiple choice permitted)

- Answering surveys
- Being interviewed (face-to-face, or via telephone/video call)
- Providing my opinion on the design of a research project (e.g. developing the project aims and outcomes)

Telling researchers how to get participants on board

Discussing with researchers how to measure success of a project

- Getting updates on how the research is going
- Discussing with researchers how to share the research with the community
- Reviewing project documents to share with the community
- Advising how research findings can be put into action
- Other [open text box]
- Unsure
- No
  1. **I would like to receive a summary of the findings from this survey**
- Yes
- No
  1. **If you selected YES to:**

**- Being recontacted for future research involvement and/or;**

**- Receiving a summary of survey findings**

**Please provide us with your email address**

[open text box]

Thank you for taking the time to complete this survey.
