# Supplementary material for: “I just don’t know enough”: Australian perspectives on community involvement in health and medical research
Source: Res Involv Engagem. 2024 Nov 28;10:126. doi: 10.1186/s40900-024-00633-8 (PMC11603817; doi:10.1186/s40900-024-00633-8)
Supplement: Supplementary file 2 — Supplementary Material 2 [file 40900_2024_633_MOESM2_ESM.docx]

**GRIPP2 Checklist**

| **Section and topic** | **Item** | **Reported on page No** |
| --- | --- | --- |
| 1: Aim | Report the aim of PPI in the study | p4, line 88-91 |
| 2: Methods | Provide a clear description of the methods used for PPI in the study | p8-9, line 174-201 |
| 3: Study results | Outcomes—Report the results of PPI in the study, including both positive and negative outcomes | p4, line 92-97  p8, line 181-190  p19-20, line 400-421 |
| 4: Discussion and conclusions | Outcomes—Comment on the extent to which PPI influenced the study overall. Describe positive and negative effects | p20-21, line 428-442 |
| 5: Reflections/critical perspective | Comment critically on the study, reflecting on the things that went well and those that did not, so others can learn from this experience | p22, line 465-478 |
